# MicroRNAs Are Key Molecules Involved in the Gene Regulation Network of Colorectal Cancer

**DOI:** 10.3389/fcell.2022.828128

**Published:** 2022-04-08

**Authors:** Fangfang Yang, Guoyun Xuan, Yixin Chen, Lichao Cao, Min Zhao, Chen Wang, Erfei Chen

**Affiliations:** ^1^ Key Laboratory of Resource Biology and Biotechnology in Western China, Ministry of Education, Northwest University, Xi’an, China; ^2^ Provincial Key Laboratory of Biotechnology of Shaanxi Province, Northwest University, Xi’an, China; ^3^ State Key Laboratory of Cancer Biology and National Clinical Research Center for Digestive Diseases, Xijing Hospital of Digestive Diseases, The Fourth Military Medical University, Xi’an, China

**Keywords:** colorectal cancer, miRNA, biomarker, gene regulation, signaling pathway

## Abstract

Colorectal cancer (CRC) is one of the most common types of cancer and one of the leading causes of mortality worldwide. MicroRNAs (miRNAs) play central roles in normal cell maintenance, development, and other physiological processes. Growing evidence has illustrated that dysregulated miRNAs can participate in the initiation, progression, metastasis, and therapeutic resistance that confer miRNAs to serve as clinical biomarkers and therapeutic targets for CRC. Through binding to the 3′-untranslated region (3′-UTR) of target genes, miRNAs can lead to target mRNA degradation or inhibition at a post-transcriptional level. During the last decade, studies have found numerous miRNAs and their potential targets, but the complex network of miRNA/Targets in CRC remains unclear. In this review, we sought to summarize the complicated roles of the miRNA-target regulation network (Wnt, TGF-β, PI3K-AKT, MAPK, and EMT related pathways) in CRC with up-to-date, high-quality published data. In particular, we aimed to discuss the downstream miRNAs of specific pathways. We hope these data can be a potent supplement for the canonical miRNA-target regulation network.

## Introduction

Colorectal cancer (CRC) remains one of the leading causes of related death worldwide. It is estimated that over 1.9 million new colorectal cancer (including anus) cases and 935,000 deaths occurred in 2020, representing about 1 in 10 cancer cases and deaths (Sung et al., 2021). Overall, colorectal cancer ranks third in terms of incidence and second in terms of mortality. Three main carcinogenic pathways determine CRC molecular profile: chromosomal instability (CIN), microsatellite instability (MSI), and CpG island methylator phenotype (CIMP) pathways (Pino and Chung, 2010). The accumulation of various genetic and epigenetic changes in colonic epithelial cells is a crucial factor driving the initiation, progression, and metastasis of CRC (Okugawa et al., 2015). In addition to canonical *APC*, *KRAS* and *TP53* mutations in CRC, epigenetic changes also play a central role in the pathogenesis of CRC.

MicroRNAs (miRNAs) are non-coding RNAs that suppress mRNA expression through complementary base pairing. In most instances, miRNAs are transcribed by RNA polymerase II to form primary miRNAs (pri-miRNAs), and subsequently cleaved into precursor miRNAs (pre-miRNAs) processed by DROSHA and DCGR8. EXPO5 mediates the export of these pre-miRNAs to the cytoplasm for further processing by DICER, which cleaves pre-miRNAs to form mature miRNAs of approximately 20 nucleotides in length. One of the strands of mature miRNA (guide strand) gets incorporated into RISC involving DICER and AGO2 to bind to target genes (Rani and Sengar, 2022; Du and Palczewski, 2022). During the last decades, miRNAs have been shown to play an essential role in cancer progression (Rupaimoole and Slack, 2017). At all stages of CRC development, miRNAs can influence cancer-related pathways by suppressing corresponding targets at the post-transcriptional level. Single miRNA alone or panel miRNAs have been shown as a potential class of valuable biomarkers (Jung et al., 2020) (Table 1). Among these miRNAs, miR-21, and miR-92a are two well-characterized oncogenic miRNAs that are identified as the most promising biomarkers for CRC early detection and prognosis. The regulation and function studies of these miRNAs were subsequently investigated, which expanded our understanding of the sophisticated mechanism of CRC pathogenesis (Pu et al., 2019). MiRNAs exert their function (proliferation, invasion, or epithelial-mesenchymal transition) by targeting specific genes involved in cancer-related signaling pathways, including the Wnt/β-catenin, PI3K/AKT, TGF-β/Smads, and EGFR pathways (Niu et al., 2021a). However, since a single miRNA can target multiple genes, and one target gene could harbour tens of hundreds of binding miRNAs (Table 2), the complex miRNA-mRNA regulatory network remains to be fully illustrated (Okugawa et al., 2015).

**TABLE 1:** List of most promising miRNA biomarkers in Colorectal Cancer.

Biomarker type	Sample type	miRNA	References
Diagnosis	Blood	miR-21, miR-92a, miR-20a, miR-29a, miR-223	(Huang et al., 2010; Du et al., 2014; Zheng et al., 2014; Yamada et al., 2015; Chang et al., 2016; Moody et al., 2019)
	Stool	miR-21, miR-17–92 cluster, miR-20a, miR-135b, miR-223, miR-421/miR-27a	(Koga et al., 2010; Wu et al., 2012; Rashid et al., 2020; Duran-Sanchon et al., 2021)
Prognosis	Blood	miR-21, miR-31, miR-34a, miR-155, miR-200 family	(Toiyama et al., 2013; Chen et al., 2014; Schou et al., 2014; Maierthaler et al., 2017; Yuan et al., 2017)
	Tissue	miR-21, miR-31, miR34a, miR-224, miR-92a	(Siemens et al., 2013; Zhang et al., 2013; Gao et al., 2015; Slattery et al., 2015; Ling et al., 2016; Mima et al., 2016)
Prediction of response to treatment	Blood/Tissue	miR-21, miR-31, miR-143/-145, miR-106	(Schetter et al., 2008; Drebber et al., 2011; Kjersem et al., 2014; Lopes-Ramos et al., 2014; Igarashi et al., 2015; Caramés et al., 2016)

**TABLE 2:** Potential targets of dysregulated miRNAs of CRC.

miRNAs	Dysregulation in CRC	Potential targets	Function	Pathway	Ref
miR-135a/b	up-regulated	APC	potential noninvasive diagnostic marker	Wnt	(Nagel et al., 2008; Okugawa et al., 2015)
miR-142-5p	up-regulated	SDHB	proliferation and apoptosis		(Liu et al., 2017; Ghafouri-Fard et al., 2021)
miR-142-3p	up-regulated	LGR5, IL-6, ABCG2, Numb, CTNNB1	tumor suppressor	Wnt, Notch	(Li and Li, 2018; Huang et al., 2020; Yadav et al., 2020)
miR-21	up-regulated	PDCD4, TPM1, PTEN, CCL20, hMSH2, PGE2	non-invasive biomarker; cell proliferation and tumor metastasis	PI3K-Akt, Wnt, P53	(Vicinus et al., 2012; Tili et al., 2013; Shi et al., 2016; Monteleone et al., 2019; Bautista-Sánchez et al., 2020; Ghafouri-Fard et al., 2021; Huang et al., 2021)
miR-92a	up-regulated	AQP8, BCL-2, KLF4, TGF-β, PTEN, p21, DKK3, SMAD7	a blood and stool-based biomarker	TGF-β, PI3K-Akt, Wnt.P53	(Chen et al., 2018; Fukada et al., 2020; Ghafouri-Fard et al., 2021)
miR-20a	up-regulated	Foxj2, PTEN, WTX	diagnostic biomarker	PI3K-Akt, Wnt	(Moody et al., 2019; Zhu et al., 2019)
miR-155	up-regulated	AXIN1, TCF4, PTEN	oncogene; cell survival and growth	Wnt	(Prossomariti et al., 2018; Huang et al., 2021)
miR-224	up-regulated	SMAD4, GSK3β, SFRP2, CDH1	cancer metastasis; noninvasive biomarkers	TGF-β	(Li et al., 2016a; Ling et al., 2016; Huang et al., 2021)
miR-223	up-regulated	RASA1	tumor suppressor; carcinogenesis, development, progression, and metastasis	MAPK, Ras	(Li et al., 2014; Sun et al., 2015)
miR-27a	up-regulated	FOXJ3, SMAD4, FAM172A	diagnostic and prognostic Biomarkers	Wnt, Hippo, MAPK, FoxO	(Xu et al., 2017; Barisciano et al., 2020; Barisciano et al., 2021)
miR-200 family (miR-141, miR-200a, miR-200b, miR-200c and miR-429)	up-regulated	RASSF2, ZEB1, ETS1, FLT1, E-cadherin, β-catenin	prognostic biomarker	Wnt	Hur et al., 2013; Yu et al., 2019)
miR-92a-3p	up-regulated	PTEN, P63, DUSP10, ITGA5, BMPR2	oncogene	PI3K-Akt, FoxO, P53	(Okugawa et al., 2015; Ahmadi et al., 2016)
miR-675-5p	up-regulated	RB	new therapeutic target; cell growth and malignant transformation	Wnt	(Tsang et al., 2010; Saieva et al., 2020)
miR-496	up-regulated	RASSF6	tumor progression and metastasis	Wnt	Wang et al., (2020a)
miR-346-5p	up-regulated	FBXL2	therapeutic target	Wnt	Pan et al., (2020)
miR-203	up-regulated	EIF5A2, BIRC5 (survivin), ATM	tumor suppressor	Wnt	Nagel et al., (2008)
miR-146a	up-regulated	Numb, c-met	tumor suppressor	Wnt	(Hwang et al., 2014; Bleau et al., 2018)
miR-135b	up-regulated	DAPK1, APC, TGFBR2	noninvasive diagnostic markers; proliferation and migration	Wnt, MAPK, FoxO, TGF-β	(Valeri et al., 2014; Huang et al., 2021)
miR-301a	up-regulated	TGFBR2	proliferation, migration and invasion	Wnt, MAPK, FoxO, TGF-β	Zhang et al., (2014)
miR-19b	up-regulated	SMAD4	proliferation and mediates	Wnt, Hippo, FoxO, TGF-β	Jiang et al., (2017)
miR-1269	up-regulated	SMAD7, HOXD10	potential marker	TGF-β	Bu et al., (2015)
miR-4775	up-regulated	SMAD7	metastasis and recurrence	TGF-β, Hippo	Zhao et al., (2017)
miR-581	up-regulated	SMAD7	metastasis; therapeutic target	TGF-β, Hippo	Du et al., (2020)
miR-4260	up-regulated	MCC, SMAD4	therapeutic target; proliferation, migration	PI3K/AKT	Xiao et al., (2017)
miR-425-5p	up-regulated	GSK3B, MAP3K5	Oncogene	PI3K/AKT	Angius et al., (2019)
miR-335	up-regulated	ZEB2, RASA1	tumor suppressor	EMT	(Sun et al., 2014; Sun et al., 2021)
miR-34a	down-regulated	MYC, SIRT1	biomarker, autophagy and malignancy	Notch, AKT, p53	(Meng et al., 2021; Zhao et al., 2022)
miR-29a	down-regulated	KLF4, PTEN, STAT3	noninvasive biomarkers; cell invasion	PI3K-Akt, JAK/STAT3	(Tang et al., 2014; Zhu et al., 2016; Wang et al., 2020b)
miR-143	down-regulated	MYO6	tumor suppressor		Luan et al., (2020)
miR-145	down-regulated	c-Myc, ERK5, SOX9	proliferation, migration and invasion	PI3K/AKT	(Ibrahim et al., 2011; Xu et al., 2018)
miR-31	down-regulated	RASA1, CDKN2B	development and progression	MAPK, Ras	(Kent et al., 2016; Xia et al., 2019)
miR-150	down-regulated	β-catenin	CRC tumorigenesis	Wnt	(He et al., 2020; Ghafouri-Fard et al., 2021)
miR-150-5p	down-regulated	CTNNB1	tumor suppressor; cell proliferation, migration, invasion and angiogenesis	Wnt, Hippo	(Aherne et al., 2015; Zhou et al., 2020)
miR-144-3p	down-regulated	BCL6	proliferation; prognostic and therapeutic target	Wnt, FoxO	Sun et al., (2020)
miR-377-3p	down-regulated	ZEB2, XIAP	proliferation and migration; tumorigenesis and metastasis	Wnt	Huang et al., (2020)
miR-532-3p	down-regulated	ETS1, TGM2	tumor suppressor	Wnt	Gu et al., (2019)
miR-455-3p	down-regulated	HSF1	tumor suppressor; proliferation		Song et al., (2020)
miR-139-5p	down-regulated	NOTCH1, AMFR, ZEB1, CTNNB1	migration and invasion	Wnt, NOTCH	(Song et al., 2014; Du et al., 2020)
miR-187	down-regulated	SOX4, NT5E, PTK6	tumor suppressor; proliferation and migration; prognostic and therapeutic biomarker	TGF-β	Zhang et al., (2016)
miR-375	down-regulated	YAP1, SP1	molecular biomarker	Hippo	Xu et al., (2019)
miR-532	down-regulated	IGF-1R	proliferation, metastasis	PI3K/AKT	Song et al., (2018)
miR-125a-3p	down-regulated	FUT5, FUT6	migration, invasion and angiogenesis	PI3K/Akt	Liang et al., (2017)
miR-4689	down-regulated	KRAS, AKT1	therapeutic agent	PI3K/Akt, Ras	Hiraki et al., (2015)
miR-195	down-regulated	Notch2, YAP1, IGF1R, RAF-1	tumor suppressor	PI3K/Akt, Notch	(Ye et al., 2016; Sun et al., 2017; Lin et al., 2019)
miR-192	down-regulated	ZEB2, Bcl-2, VEGFA	biological markers	EMT	Geng et al., (2014)
miR-218	down-regulated	ZEB2, N-cadherin, CTGF	invasion and metastasis	EMT	(Mudduluru et al., 2015; Lun et al., 2018)

In this review, we provide an overview of dysregulated miRNAs in CRC and highlight the promising miRNAs biomarkers involved in the CRC-related signaling pathways. We present a systematic search of up-to-date studies with high-quality profiling of the miRNAs involved in the progression or metastasis of CRC, mainly published from January 2019 to January 2022. In particular, we focus on the downstream miRNAs of specific pathways. We hope these data can be a potent supplement for the canonical miRNA-target regulation network.

### Wnt Signaling Pathway

**FIGURE 1:**
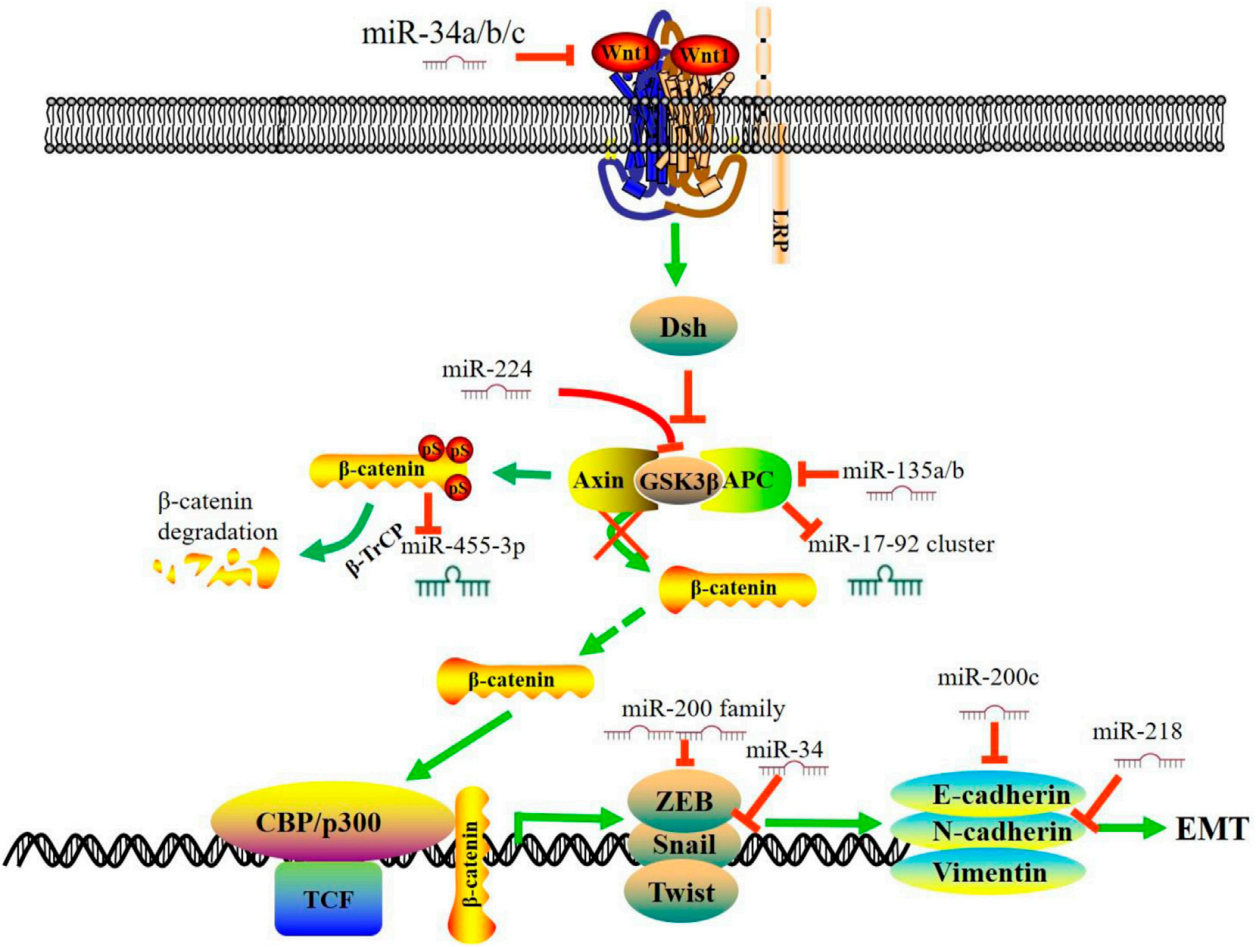
MiRNAs regulate Wnt/β-catenin signaling pathway and EMT in CRC. In the absence of Wnt, β-catenin forms a complex with APC, GSK3 and Axin. The β-catenin is phosphorylated by GSK3β and targeted for ubiquitin-dependent proteasomal degradation. This figure summarizes the interaction between miRNAs and the Wnt/β-catenin signaling pathway in CRC and a subset of downstream miRNAs that are involved in the EMT process. During the process of EMT, the expression of mesenchymal markers, including N-cadherin and vimentin, is up-regulated, and the expression of the epithelial markers, including E-cadherin and ZO-1, is down-regulated. miR, microRNA; EMT, epithelial-to-mesenchymal transition; APC, Adenomatous Polyposis Coli; TCF, T-cell factor.

In different stages of development and particular cellular conditions, the canonical Wnt pathway is triggered by attaching the Wnt ligands to specific receptors. Stimulation of the canonical Wnt signaling leads to the inhibition of the GSK-3β, thus triggering the accumulation of stabilized β-catenin in the cytoplasm. β-catenin translocates into the nucleus and interacts with T-cell factor/lymphocyte enhancer factor (TCF/LEF) and subsequently loads Brg-1 and p300/CBP proteins onto promoters, thereby regulating the transcription of multiple target genes involved in cell proliferation, differentiation, and apoptosis (Azbazdar et al., 2021). Studies reported more than 80% of CRC samples have been detected with Wnt pathway activation, mainly caused by adenomatous polyposis coli (*APC*) or β-catenin (*CTNNB1*) coding gene mutations. Evidence has illustrated that the aberrant expression of microRNAs upstream of the Wnt cascade could be involved in CRC initiation and progression (Figure 1). As key oncogenes in the Wnt pathway, miR-135a/-135b directly target the 3′-UTR of APC to suppress its expression and induce downstream Wnt pathway activation (Nagel et al., 2008). Stool miR-135b expression is dramatically up-regulated in CRC patients. It targets the mRNA of ZNRF3, a zinc and ring finger protein that acts as a tumor suppressor in the intestinal stem cell zone by inhibiting the Wnt signaling pathway. MiR-135b may be a promising non-invasive biomarker for diagnosing CRC patients with TNM stage-III/IV (Li et al., 2020). Decreased levels of miR-142 and miR-150 were detected in CRC patients. *In vitro* function assays suggest that these two miRNAs could directly bind to CTNNB1 and thus inhibit CRC tumorigenesis (He et al., 2020; Liu et al., 2020). MiR-34a/b/c can directly act and inhibit the effector molecules of the Wnt signaling pathway, including WNT1, WNT3, LRP6, and CTNNB1 (Kim et al., 2011; Jiang and Hermeking, 2017), while inhibiting the WNT signaling pathway. MiR-224/GSK3β axis, identified by bioinformatics and luciferase function studies, can lead to the activation of Wnt/β-catenin signaling and the nuclear translocation of β-catenin (Li et al., 2016a). Saieva *et al* also reported that inhibition of the hypoxia-induced miR-675-5p could hamper the nuclear localization of β-catenin by regulating GSK-3β activity (Saieva et al., 2020). The ectopic expression of miR-92a-3p up-regulates cell cycle- and mitosis-related gene expression while down-regulates adhesion-related gene expression in endothelial cells by targeting Wnt regulatory protein DKK3 and Claudin-11 (Yamada et al., 2019). In addition, recent studies have also reported several miRNAs indirectly involved in Wnt pathway-mediated tumor progression and metastasis, such as elevated miR-496 (Wang et al., 2020a), miR-346-5p (Pan et al., 2020), and miR-203 (Peng et al., 2020), and down-regulated miR-144-3p (Sun et al., 2020), miR-377-3p (Huang et al., 2020), miR-532-3p (Gu et al., 2019), and miR-200c (Cong et al., 2021).

Emerging studies have revealed a subset of miRNAs downstream of the Wnt signaling pathway. Activation of Wnt pathway alters the expression of these miRNAs and triggers the malignant phenotype. Heat shock transcription factor 1 (HSF1) is overexpressed in CRC and is vital for β-catenin to promote CRC development. β-catenin overexpression suppresses miR-455-3p generation to stimulate m6A modification. The translation of HSF1 mRNA is impaired after β-catenin depletion, accompanied by the up-regulation of miR-455-3p (Song et al., 2020). In KRAS-mutant cells, aberrantly activated Wnt signaling represses miR-139-5p through Transcription Factor 4 (TCF4), which forms a miR-139-5p/Wnt signaling feedback loop (Du et al., 2020). The APC and β-catenin protein could regulate miR-17–92 cluster, and the loss of APC can lead to the activation of β-catenin, thus activating the miR-17–92 promoter (Li et al., 2016b). Snail, a downstream effector and target of Wnt, induces the expression of miR-146a through the β-catenin-TCF4 complex. Meanwhile, miR-146a can target Numb to stabilize β-catenin, forming a feedback loop to maintain Wnt activity (Hwang et al., 2014).

### TGF-β Signaling Pathway

**FIGURE 2:**
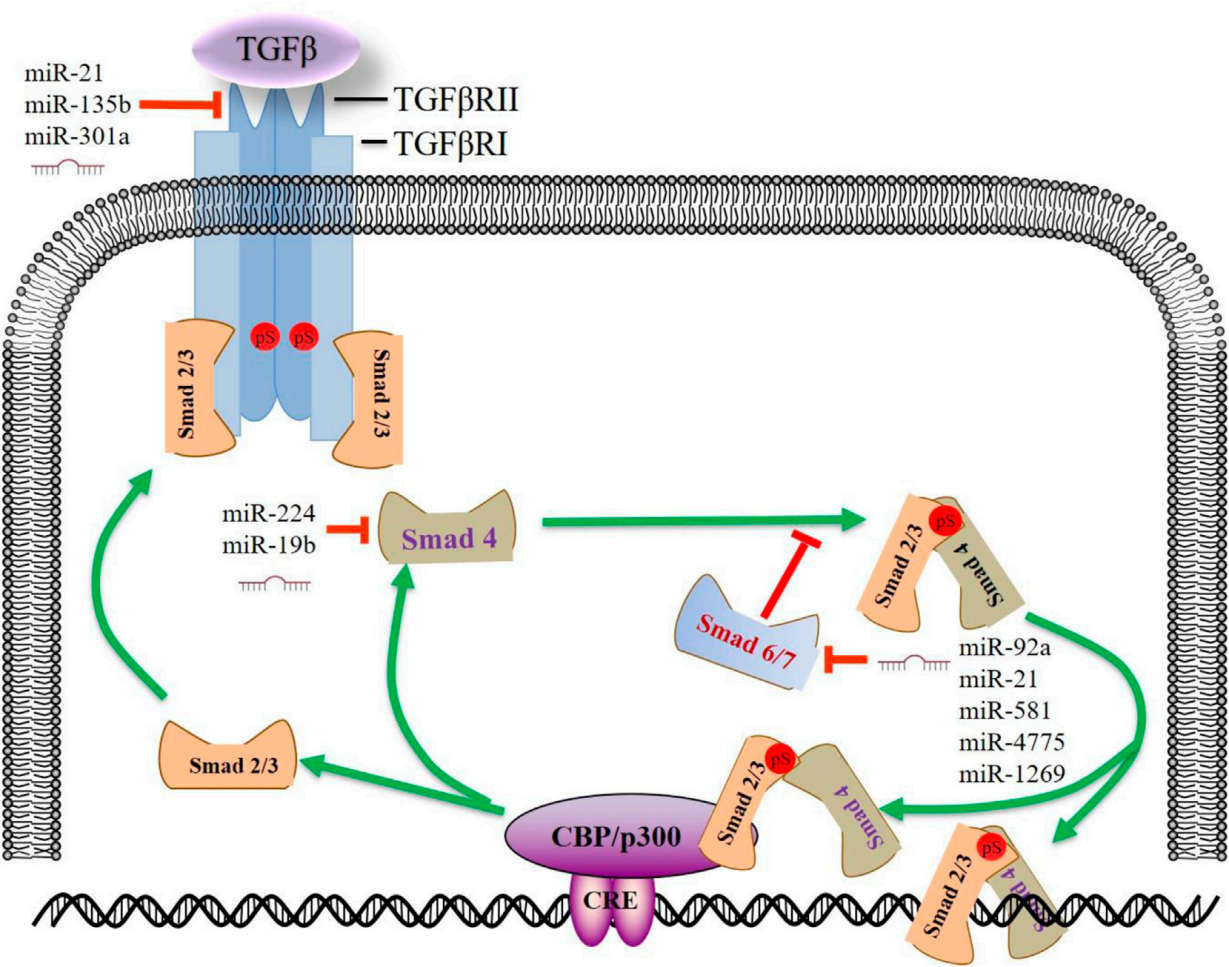
MiRNAs regulate the TGF-β signaling pathway in CRC. Upon TGF-β stimulation, activated TGFBR2 binds to and phosphorylates TGFBR1. Activated TGFBR1 phosphorylates SMAD2/SMAD3, enabling SMAD2/SMAD3 to bind SMAD4 and subsequent activation or repression of TGF-β responsive genes. Some miRNAs enhance the TGF-β signaling by targeting SMAD7, forming a positive feedback loop. This figure summarizes some prominent miRNAs implicated in CRC progression by targeting TGF-β signaling pathway. miR, microRNA; CRE, cAMP response element; TGFβ, transforming growth factor β; SMAD, SMAD Family Member; TGFβR, transforming growth factor β receptor.

The transforming growth factor—beta (TGF-β) signaling depends on Smad family proteins. Active TGF-β1 at the cell surface mediates signaling through the TGF-β type I and type II receptors (TβRI and TβRII) which are active serine/threonine kinases. The TGF-β/receptor complex subsequently phosphorylates receptor-activated Smads (R-Smads). R-Smads translocate to the nucleus and regulate the transcription of target genes (Tecalco-Cruz et al., 2018). TGF-β signaling pathway plays an essential role not only in normal colonic tissue homoeostasis but in CRC progression (Farooqi et al., 2019). This canonical pathway exerts a dual role in the carcinogenesis of various cancer types. In the early stages, the TGF-β pathway acts as a tumor suppressor, while in the advanced stages, and it enhances the malignant phenotype (Luo et al., 2019; Niu et al., 2021b; Vanpouille-Box and Formenti, 2018). Studies reported that more than 90% of microsatellite instability (MSI) tumors are affected by frameshift mutations in the TGFBR2 gene, leading to impaired receptor expression, and abrogated downstream signaling (Fricke et al., 2019). Due to the relatively long 3′-UTR of TGFBR2 (∼9.2 k), studies reported that many onco-miRNAs are involved in TGFBR2 regulation. MiR-21, miR-135b, and miR-301a exert their tumor-promoting role in proliferation, migration, and invasion in CRC cells by targeting TGFBR2 (Figure 2) (Yu et al., 2012; Zhang et al., 2014).

In the SMAD-dependent pathway, phosphorylated Receptor-regulated-SMADs (R-SMADs, SMAD2 or 3) form functionally active complexes with joint partner SMAD4 and move into the nucleus to regulate a myriad of target genes (Itatani et al., 2019). A recent study showed that miR-224 could promote CRC metastasis, at least in part, through the regulation of tumor suppressor SMAD4. MiR-224 expression in primary CRC, alone or combined with SMAD4, may have prognostic value for the survival of patients with CRC (Ling et al., 2016). An elevated level of miR-19b was an independent prognostic factor associated with CRC patients’ survival. MiR-19b/SMAD4 promotes cell proliferation and mediates resistance to oxaliplatin-based chemotherapy (Jiang et al., 2017).

SMAD7, a negative regulator of the TGF-β/SMADs pathway, binds to TβR1 and competes with SMAD2/3, thus preventing the phosphorylation of R-SMADs (Stolfi et al., 2020). Meanwhile, SMAD7 can enhance the ubiquitination and proteasome-mediated degradation of TβR1 by SMURF1/2. In the last 5 years, the role of miRNA/SMAD7/TGFβ axis in CRC metastasis has been broadly discussed. MiR-21 (Jiao et al., 2017), miR-1269 (Bu et al., 2015), miR-4775 (Zhao et al., 2017), and miR-581 (Zhao et al., 2020) were uniquely up-regulated and negatively correlated with SMAD7 expression in metastatic CRC samples. Function assays confirmed that these miRNAs exert oncogenic roles only in regulating migration and invasion. Similarly, these miRNAs enhance TGF-β signaling by targeting SMAD7, forming a positive feedback loop. Based on the binding capacity and correlation analysis, bioinformatics also revealed that SMAD7 is among the top target of miR-92a, the blood and stool-based biomarker of CRC (Chen et al., 2018) (Table 1).

On the other hand, activation of the TGFβ pathway can trigger the alteration of some downstream miRNAs. MiR-187, a downstream effector of the TGFβ pathway, suppresses Smad-mediated epithelial-mesenchymal transition in CRC (Zhang et al., 2016). MiR-187 directly targets SOX4, NT5E, and PTK6, which are essential upstream effectors of the Smad pathway, resulting in a sustained TGFβ activation in cancer progression. Furthermore, TGFBR2 deficiency in HCT 116 cells leads to the down-regulation of miR-381-3p, which is expected to be of functional relevance and may contribute to MSI-specific tumor characteristics (Fricke et al., 2019). In addition, we analyzed the GEO dataset GSE53338 (TGF-β knock-down cells) and found that CRC biomarkers miR-31, miR-145, and miR-20a were down-regulated in the TGF-β knock-down cells. These results suggest that activation of TGF-β/SMADs could also influence Smad-dependent downstream miRNAs, thus contributing to CRC progression.

### PI3K-AKT Pathway

**FIGURE 3:**
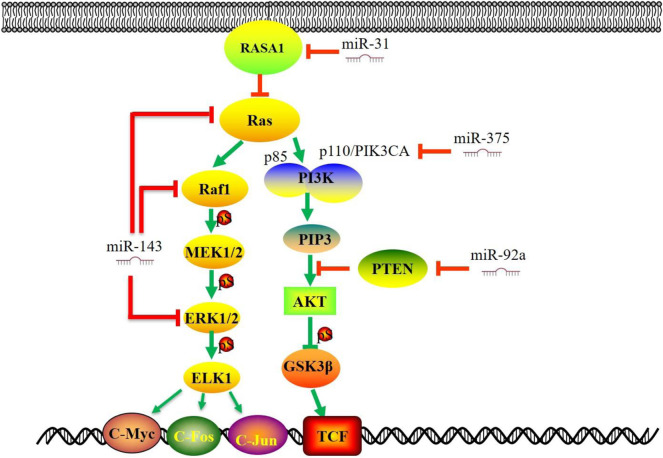
MiRNAs regulate Ras-Raf-MAPK and PI3K/AKT signaling pathways in CRC. Several studies have demonstrated that miRNAs target the key molecules in the MAPK signaling pathway (RAS, RAF, ERK, MEK5, ELK1 etc.). The diversity of alterations in the PI3K/AKT signaling pathway (p110, p85, AKT, PTEN, etc.) provides multiple molecular targets for therapy and raises the challenge of CRC. This figure summarizes that some significant miRNAs are involved in CRC progression by targeting critical molecules in MAPK and PI3K/AKT signaling pathway. miR, microRNA; PI3K, phosphatidylinositol 3-kinase; PTEN, phosphatase and tensin homolog; GSK3β, Glycogen Synthase Kinase 3 Beta; RASA1, RAS P21 Protein Activator 1; TCF, T-cell factor.

The PI3K/Akt is a pathway that can promote cell survival and growth. Lipid kinase PI3K phosphorylates phosphatidylinositol 4,5-bisphosphate (PIP2) to generate phosphatidylinositol 3,4,5-trisphosphate (PIP3), which in turn phosphorylates protein kinase B (AKT) to cause a cascade of responses of cellular function. PIP3 can be dephosphorylated back to PIP2 by the lipid phosphatase PTEN (Hoxhaj and Manning, 2020). Activation of the PI3K/AKT/mTOR signaling is a prominent feature of human cancers (Hoxhaj and Manning, 2020; Fruman et al., 2017). PI3K/AKT signaling regulates cell survival and proliferation (Cantley, 2002; Ediriweera et al., 2019). Consequently, molecules in this pathway (p110, p85, AKT, mTOR, and PTEN, etc.) provide multiple novel targets for therapy (Mayer and Arteaga, 2016). MiRNAs could directly target the key molecules in the PI3K signaling pathway to promote the progression of CRC (Figure 3). MiR-21 expression is up-regulated in CRC and functions as an oncomir in CRC through PTEN (Xiong et al., 2013; Liu et al., 2019). Beyond that, miR-92a directly targets 3′-UTR of PTEN. The up-regulation of miR-92a inhibits the expression of PTEN, thus activating the PI3K/AKT signaling pathway and promoting CRC cell proliferation and lymph node metastasis (Ke et al., 2015; Chen et al., 2018). In a meta-analysis of cancer genome sequencing results, PIK3CA and PTEN are highly mutated genes in most human cancers (Lawrence et al., 2014). Herein, miR-375 represses CRC tumorigenesis by targeting PIK3CA by inactivating the PI3K/AKT signaling pathway. Moreover, miR-125a-3p directly inhibits FUT5 (fucosyltransferase) and FUT6, downstream targets of the PI3K/AKT signaling pathway in CRC. (Liang et al., 2017). This evidence indicates that the up-regulation of the PI3K/AKT signaling pathway is an essential aspect of miRNAs in CRC pathogenesis.

### Ras-Raf-MAPK Signaling Pathway

The Ras-Raf-MAPK signaling pathway plays a central role in the survival and development of normal and tumor cells. (Downward, 2003; Collisson et al., 2014). Extracellular ligand (such as growth factor) binds to receptor tyrosine kinase (RTKs), which results in the recruitment of small G-proteins (RAS) to the cell membrane. In turn, RAS-GTP directly binds to RAF protein, thus phosphorylating MEK and ERK, followed by the activation of the mitogen-activated protein kinase (MAPK) pathway. (Figure 3). The MAPK pathway is a downstream effector of many growth-factor receptors, including the epidermal growth factor receptor (EGFR) (Fang and Richardson, 2005). Recent studies have shown that down-regulation of miR-143 and, or the up-regulation of miR-31 could trigger the RAS-RAF–MAPK pathway to enhance proliferation and modulating treatment responses (Sun et al., 2013; Akao et al., 2018). Furthermore, the expression of miR-143 was decreased in CRC specimens, and KRAS was a target gene of miR-143 (Luo et al., 2020). MiR-31 directly targets RAS p21 Protein Activator 1 (RASA1), a negative regulator of the RAS pathway, and modulates invasion behavior through interaction with p190RhoGAP and its downstream proteins, which plays essential roles in cell proliferation and differentiation (Buday and Downward, 2008; Sun et al., 2013). MiR-425-5p might participate in the pathogenesis of KRAS-mutated CRC and contribute to this subcategory of CRC (Angius et al., 2019). As a negative regulator, miR-4689 potently suppresses the oncogenic KRAS-driven EGFR signaling pathways through the direct inhibition of KRAS and AKT1. The loss of miR-195 lessens its binding and inhibitory effect to RAF-1, thus promoting colorectal cancer cell proliferation and cell survival (Ye et al., 2016).

The activation or mutation of BRAF and, or KRAS can trigger the abnormal expression of downstream miRNAs. Tumors with BRAF and KRAS mutations showed significantly elevated levels of miR-31 expression (Lundberg et al., 2018). MiR-193a, one of the candidate miRNAs in BRAF-mutated cells, acts as a tumor suppressor by regulating multiple cancer-related genes and affects cellular sensitivity to MAPK-related pathway inhibitors, such as BRAF inhibitors, MEK inhibitors, and, or anti-EGFR antibodies (Hiraide et al., 2021). MiR-139-5p could serve as a novel regulator of the crosstalk between the Ras and Wnt signaling pathways. Aberrantly activated Wnt signaling in KRAS-mutant cells represses miR-139-5p through TCF4, forming a double negative feedback loop of mir-139-5p/Wnt signal (Du et al., 2020). Mutant KRAS functions as a broad regulator of the EGFR signaling cascade by inhibiting miR-4689, negatively regulating both the RAS/MAPK and PI3K/AKT pathways (Hiraki et al., 2015). Therefore, targeting miRNAs that play a critical role in the Ras-Raf-MAPK signaling pathway might be an alternative therapy in CRC patients with aberrant Ras-Raf-MAPK signaling.

### CRC Metastasis-Related Pathways

Epithelial-to-mesenchymal transition (EMT) is a process which cells lose their epithelial characteristics while gain mesenchymal properties, which has a critical role in tumor initiation, progression, and metastasis (Tsai and Yang, 2013; Dong et al., 2020; Taki et al., 2021). Growing evidence from preclinical studies has shown that some EMT biomarkers could serve as potential therapeutic targets (Zhang et al., 2021). The mesenchymal markers, including N-cadherin and Vimentin, are up-regulated, while the epithelial markers (E-cadherin, EpCAM, and ZO-1) are down-regulated during the EMT process (Loh et al., 2019; Satelli and Li, 2011). In addition, the transcription factors, such as Twists, Snails, Slug and ZEBs, also participate in this essential program. (Diepenbruck and Christofori, 2016; Mittal, 2018). Accordingly, miRNAs can regulate EMT by inhibiting the genes involved in EMT in CRC (Figure 1). The miR-200 family is a group of microRNAs with five members: miR-141, miR-200a, miR-200b, miR-200c, and miR-429, which target the EMT signaling pathway (O'Brien et al., 2018). In CRC patients, decreased expression of miR-429, miR-200a, and miR-200c is associated with worse survival. Gollavilli et al. reported that miR-200b/c low expressing cells could form a significantly lower number of liver metastasis in nude mice, which is in line with the partial EMT phenotype. In addition, a Seahorse metabolic analysis showed a drastic reduction of OXPHOS ability which further explain the loss of growth observed in miR-200b/c low-expressing cells. MiR-205-5p functions as a tumor suppressor and enhanced expression of miR-205-5p can significantly raise the levels of E-cadherin by targeting ZEB1 (Gulei et al., 2018). Furthermore, miRs-192, -218, and -335 (Figure 1) target ZEB2, and miR-218 also targets N-cadherin to promote EMT (Geng et al., 2014; Mudduluru et al., 2015). Transcription factors, including Snails and ZEBs, also regulate downstream miRNAs to affect the CRC malignant phenotype. In HT-29 cells, Snail overexpression repressed the level of miR-192 and miR-194 and increased the levels of miR-205 and let-7i (Przygodzka et al., 2019). Wang et al. confirmed that the chimeric [SNAIL/miR-34] [ZEB/miR-200] system might be the core axis for the EMT process (Wang et al., 2018).

Epidemiological and experimental studies have revealed that inflammatory signaling pathways are also involved in CRC metastasis (Świerczyński et al., 2021). NF-κB and STAT3 pathways are two critical regulators of inflammation. MiR-21 is also involved in the regulation of NF-κB and MyD88. MiR-21 reduces the tumor burden and decreases the expression of pro-inflammatory cytokines. Meanwhile, by depleting miR-21 in tumors, the PDCD4 expression increased, while the expression of STAT3, and BCL2 decreased (Bakirtzi et al., 2011; Shi et al., 2016). MiR-221 and miR-222 can also activate NF-κB and STAT3 by indirectly modulating their protein stability through miR-221/222-mediated positive feedback loops (Liu et al., 2014). IL-6/STAT3/MMPs signaling induces EMT and metastasis in colorectal cancer. An active IL-6R/STAT3/miR-34a loop is necessary for this program in CRC cells and this could affect nodal and distant metastasis in CRC patients (Rokavec et al., 2014). Yang and colleagues found that miR-1301 is significantly down-regulated in CRC samples. Functionally, miR-1301 regulates CRC cell migration and invasion capacity through the STAT3/MMPs axis (Yang et al., 2021). MiR-146 can function as a negative regulator of colonic inflammation. In myeloid cells, miR-146a prevents intestinal inflammation by limiting myeloid cell-mediated inflammatory IL-17 production or inhibiting tumorigenic IL-17R signaling in intestinal epithelial cells (Garo et al., 2021).

## Summary and Future Perspective

Colorectal cancer usually requires the surgical removal of tumor tissue, chemotherapy and targeted therapy. At present, new targeted drugs are mainly aimed at the tumor angiogenesis endothelial growth factor (vascular endothelial growth factor, VEGF) and receptor (vascular endothelial growth factor receptor, VEGFR), or the epidermal growth factor receptor (EGFR) (Rio-Vilariño et al., 2021). However, more than 30% of cases are resistant to EGFR inhibitors, so it is crucial to find new therapeutic targets (Mangiapane et al., 2022). Anti-tumor immunotherapy has become another essential means of treating colorectal cancer after surgery, chemotherapy, radiotherapy, and targeted therapy. In this field, negative regulators of the immune system, which are called immune checkpoints, play a vital role in limiting anti-tumor immune responses. On this account, immune checkpoint-inhibiting agents, like those directed against cytotoxic T-lymphocyte antigen 4 (CTLA-4), programmed death-1 receptor (PD1), and its ligand PD-L1, have been developed as anti-tumor drugs, producing exciting results in preclinical and clinical studies (Ganesh et al., 2019).

Given the multiple studies on ncRNAs, miRNAs undoubtedly hold great potential as future biomarkers for the diagnosis, prognostication, and response prediction. Inhibitors targeting onco-miRNAs might be a fundamental treatment approach, at least at the hypothetical level. Despite the preclinical evidence, no clinical trials currently evaluate miRNA mimics specifically in CRC. A systematic understanding of the miRNAs’ regulatory mechanisms will explore their future clinical applications as biomarkers or their potential as therapeutic targets in CRC. In recent years, long noncoding RNAs (lncRNAs) have been demonstrated to be critical factors in colon cancer development and progression. LncRNAs can regulate gene transcription by binding miRNAs and acting as a sponge. LncRNAs, miRNAs and mRNA all together contribute to the biological processes in CRC, including metastasis, EMT, or inflammation formation. The in-depth studies in this field will provide new ideas for the diagnosis and treatment of CRC. However, the complex mechanism of LncRNA/miRNA/mRNA axis and assessing their values in CRC early detection or treatment would be a great challenge (Toiyama et al., 2018; Marcuello et al., 2019).

Taken together, this review summarized the up-to-date studies in the field of CRC-related miRNAs and the complex regulating network. We focused on miRNAs that could serve as important indicators for CRC and highlighted the dysregulated miRNAs downstream of specific pathways. This study would be helpful for further understanding of the non-coding RNA mechanism in CRC and provide new outlooks of these molecules for their utilization as targets for the treatment of CRC in the future.
